# Alcohol use disorders and related morbidity and mortality after sleeve gastrectomy and Roux-en-Y gastric bypass: a nation-wide registry study (the BAR-REGISTER)

**DOI:** 10.1038/s41366-026-02123-1

**Published:** 2026-06-17

**Authors:** Magnus Strømmen, Inger Johanne Bakken, Jorunn Sandvik, Jørgen Gustav Bramness, Christian Klöckner

**Affiliations:** 1 Centre for Obesity Research, Clinic of Surgery, St. Olavs Hospital, Trondheim University Hospital, Trondheim, Norway; 2 Department of Clinical and Molecular Medicine, Norwegian University of Science and Technology (NTNU), Trondheim, Norway; 3 Department of Chronic Diseases, Norwegian Institute of Public Health, Oslo, Norway; 4 Department of Surgery, Møre og Romsdal Hospital Trust, Ålesund, Norway; 5 Department of Health Sciences, Norwegian University of Science and Technology (NTNU), Ålesund, Norway; 6 Institute of Clinical Medicine, UiT The Arctic University of Norway, Tromsø, Norway; 7 Department of Clinical Addiction Research, Oslo University Hospital, Oslo, Norway; 8 Research Center for Substance Use Disorders and Mental Illness, Innlandet Hospital Trust, Hamar, Norway; 9 Department of Psychology, Norwegian University of Science and Technology (NTNU), Trondheim, Norway

**Keywords:** Obesity, Obesity, Epidemiology

## Abstract

**Background:**

Bariatric surgery provides notable weight loss and metabolic benefits but may also increase the risk of alcohol use disorder (AUD). We investigated whether Roux-en-Y gastric bypass (RYGB) confers higher rates of AUD and related disorders than sleeve gastrectomy (SG) and examined associated morbidity and mortality.

**Methods:**

We conducted a retrospective, population-based cohort study using data from the Norwegian Patient Registry and the Norwegian Prescription Database. A total of 17,800 patients underwent RYGB (*n* = 12,244) or SG (*n* = 5556) between 2008 and 2018. Patients with prior diagnoses related to alcohol or use of medications for AUD were excluded. Incidence rates (IR) for alcohol-related diagnoses were calculated per 1000 person-years; hazard ratios (HR) were derived comparing RYGB to SG. Morbidity was measured as number of specialized healthcare contacts, but also as number of prescriptions/defined daily doses in an exploratory model.

**Results:**

Mean postoperative follow-up was 5.7 years (RYGB) and 3.8 years (SG). By 31 December 2018, 576 patients (3.2%) had developed a new alcohol-related diagnosis – an incidence rate of 6.34 per 1000 person-years. The adjusted HR for such diagnoses was 1.69 (95% CI 1.33–2.13, *p* < 0.001) for patients undergoing RYGB compared to SG. Because mortality did not differ significantly between RYGB and SG, mortality was assessed for the cohort as a whole: patients with alcohol-related diagnoses had an adjusted HR for death of 2.08 (95% CI 1.40–3.08) relative to those without. They also recorded, on average, 5.5 additional contacts in specialist care.

**Conclusions:**

Compared with SG, RYGB was associated with a 69% higher risk of alcohol-related diagnoses. Further, given the elevated morbidity and mortality linked to these disorders, enhanced preoperative screening and long-term postoperative monitoring are warranted in modern bariatric practice.

## Introduction

Despite strong evidence that bariatric surgery effectively promotes weight loss and improves metabolic disorders [1], growing research points to an elevated risk of alcohol use disorder (AUD) associated with certain bariatric procedures. Long-term follow-up data from Sweden and Denmark indicate that patients who have undergone Roux-en-Y gastric bypass (RYGB) face a five- and seven-fold higher incidence, respectively, of AUD compared to individuals with obesity who do not undergo bariatric surgery [2, 3]. Researchers have also examined whether AUD risk varies by type of bariatric procedure. RYGB seems to be associated with a higher risk of AUD than vertical banded gastroplasty and gastric banding [3–5]. In terms of sleeve gastrectomy (SG), the most frequently performed bariatric procedure, two studies from the US found the risk of AUD to be less compared to after RYGB [6, 7]. Due to several factors, AUD is heavily underdiagnosed [8]. To increase the chances of picking up the true impact of alcohol, it is important to include also other alcohol-related morbidities.

Although the drivers of elevated postoperative AUD risk are multifaceted, pharmacokinetic changes in ethanol absorption and metabolism are likely to play a decisive role. Studies on RYGB-patients have reported a shorter time to reach peak blood alcohol concentration (BAC) [9, 10] and an increased maximum BAC [9, 11, 12], which result in a higher alcohol exposure. Pharmacokinetic studies on SG have been less consistent: some studies found similar changes as those observed after RYGB [13, 14], whereas others have concluded that SG does not influence ethanol pharmacokinetics [15, 16]. However, interpretation of the existing literature is complicated by methodological limitations in several studies, as time-to-peak and peak exposure may be difficult to infer reliably from breath-based measurements; as shown by Acevedo et al., breath sampling can misestimate the timing and magnitude of peak BAC and thereby obscure true pharmacokinetic effects [13].

We recently published results from a longitudinal, controlled study comparing the pharmacokinetics of ethanol before and after RYGB and SG. Both procedures significantly shortened the time it took to reach the peak BAC, resulted in a significantly increased maximum BAC and roughly doubled alcohol exposure, with these effects persisting throughout the 3-year observation period. Furthermore, peak BAC was reached 31% faster after RYGB than after SG, and maximum BAC was 27% higher [17]. Generally, the faster a substance produces a feeling of euphoria (i.e. reaching its peak level in the brain), the greater its addictive potential [18]. The sudden spike in intoxication that individuals can experience after undergoing bariatric surgery, even after small or moderate alcohol consumption, could be a significant risk factor for developing AUD. Although SG, like RYGB, entails substantial alterations in ethanol pharmacokinetics, these effects appear to be somewhat less pronounced after SG than after RYGB.

We previously performed a comparison of risk of AUD in SG compared to RYGB based on data from the Norwegian Patient Registry and found a hazard ratio for SG compared to RYGB to be 0.71 [19]. Although this aligns with the findings from US studies [6, 7], our results did not reach statistical significance, possibly due to relatively few SG-patients and a limited observation period.

In the present study, we performed a more extensive extraction of registry data, hypothesizing that RYGB is associated with a higher risk of AUD than SG. We also investigated the morbidity and mortality linked to postoperative AUD.

## Material and methods

We conducted a retrospective, population-based cohort study by combining data from two central registries in Norway.

### Data sources

The Norwegian Patient Registry (NPR) is a national database that covers all government-reimbursed specialist health-care services in Norway, covering both in-hospital care and out-patient clinics. From 2008 onwards, it includes an 11-digit personal identification number that allows patient tracking over time and linkage with other databases. Diagnoses are recorded according to the International Classification of Diseases, version 10 (ICD-10), and treatment procedures are registered with codes from the Nordic Medico-Statistical Committee Classification of Surgical Procedures (NCSP). The NPR also provides demographic data, information about the treating hospital, and consultation/admission dates [20].

Established in 2004, the Norwegian Prescription Database (NorPD) contains information on all prescriptions filled at pharmacies in Norway, giving good knowledge of all drugs purchased except for over the counter drugs [21].

### Incidences of alcohol-related diagnoses

A patient was defined as having a new onset alcohol-related diagnosis if:

1. They had no alcohol-related diagnosis (NPR-data from Jan 1, 2008) or alcohol-related prescription (NorPD-data from Jan 1, 2004) prior to surgery.
2. They received a first alcohol-related diagnosis and/or relevant alcohol-related prescription in the postoperative period (data up to December 31, 2018).
3. Their first alcohol-related diagnosis/prescription did not occur within 6 months of surgery.

In addition to F10*-diagnoses (mental and behavioural disorders due to alcohol), a limited number of patients were identified based on other diagnoses associated with alcohol-related conditions. These included G62.1 (alcoholic polyneuropathy), K29.2 (alcoholic gastritis), K70 (alcoholic liver disease), K86.0 (chronic pancreatitis due to alcohol, Z71.4 (counselling for alcohol use), and Z72.1 (issues related to alcohol consumption). The alcohol-relevant drug prescriptions included disulfiram, acamprosate and nalmefene (ATC-codes N07B B01, B03 and B05 in the NorPD-data).

To enhance readability, we have chosen the term “AUD+” throughout this paper to denote individuals with alcohol-related diagnoses, i.e. F10*, other alcohol-related diagnoses, or the use of medications associated with the treatment of AUD.

### Identifying the study population and cases

Approximately two thirds of bariatric surgeries in Norway are performed in public hospitals and are recorded in the NPR. We used the NPR to identify patients who underwent bariatric surgery. The initial dataset included 18,972 unique patients who had 1,294,125 contacts (outpatient consultations and hospitalizations) in the specialist health-care services in the period 2008–2018.

A total of 19,523 surgery registrations (JDF10/11 [RYGB], JDF40/41/96/97 [SG], and JFD03/04 [duodenoileal bypass with duodenal switch]) were identified. We excluded 697 patients with duplicate registrations and 905 patients who had less than 6 months of postoperative follow-up. Because of low procedure numbers, we also excluded 121 patients with the duodenoileal bypass with duodenal switch. This left 17,800 patients eligible for further analysis.

Out of 1,294,125 specialist healthcare contacts observed over the study period, 17,226 involved an alcohol diagnosis. In total, 755 patients received at least one F10 diagnosis during the observation period; 551 of these received their first F10 diagnosis postoperatively.

Next, we linked NPR data to NorPD, which contained information on 4,678,923 prescriptions for the same population. Focusing on alcohol-related ATC-codes (N07B B01/B03/B05), we identified 1391 prescriptions, corresponding to 333 potential AUD+ cases; 258 of these had their first relevant prescription postoperatively. Linking the two databases prevented misclassification of patients who would have appeared to develop an alcohol-related disorder postoperatively if only one data source had been used. After cross-checking, 595 had a new F10 diagnosis or alcohol-related prescription postoperatively. Excluding those who had onset within the first 6 months yielded 576 final AUD+ cases (493 RYGB, 83 SG). Incidence calculations were based on these subsets.

Overall prescription counts were examined as a secondary, exploratory utilization measure; due to known limitations of this outcome (e.g., dispensing practices and package sizes), these analyses are reported in the Supplementary Material.

### Analysis

We calculated AUD+ incidence rates (IR) as the number of new cases divided by person-years at risk. Crude hazard ratios (HR) were derived by dividing the IR of RYGB by the IR of SG. One-way ANOVA with Games-Howell post hoc tests was used to assess differences in diagnosis risk by age and sex, and to compare number of specialist care contacts and prescriptions. Multiple regression (enter model) predicted the number of contacts and prescriptions when controlling for age and sex. A sensitivity analysis restricting the cohort to procedures performed in 2014–2018, when both procedures were widely used, were performed as the choice of surgery changed over time. We used a significance level of *p* < 0.05 throughout and, where relevant, report 95% confidence intervals. Analyses were conducted using SPSS version 30 (IBM SPSS Statistics, IBM Corporation, Armonk, NY, USA).

## Results

In total, 17,800 patients underwent either RYGB (12,244) or SG (5556) between 2008 and 2018, corresponding to 90,798 person-years at risk from the date of surgery until December 31, 2018 (Tables 1 and 2). The average postoperative follow-up time was 5.7 years (SD 2.9) for patients with RYGB and to 3.8 years (SD 2.5) for those with SG. A slightly higher proportion of women underwent SG than men (74.7 vs 73.3%, *p* = 0.046). There was no significant age difference between the SG and RYGB groups (42.5 vs 42.7 years, *p* = 0.179).

**Table 1:** Patient demographics and postoperative observation time among patients undergoing bariatric surgery (*N* = 17,800).

	SG (*N* = 5556)	RYGB (*N* = 12,244)	
Sex, women	74.7%	73.3%	*p* = 0.046
Age at time of surgery	42.5 years (SD 11.3)	42.7 years (SD 10.4)	*p* = 0.179
Postoperative observation time	3.8 years (SD 2.5)	5.7 years (SD 2.9)	*p* < 0.001

*SG* sleeve gastrectomy, *RYGB* Roux-en-Y gastric bypass.

**Table 2:** Incidence rates per 1000 person-years (IR) and hazard ratios (HR) with associated 95% confidence intervals (CI) for patients registered with diagnoses related to alcohol following Roux-en-Y Gastric Bypass (RYGB) or Sleeve Gastrectomy (SG) in hospitals covered by the Norwegian National Insurance Scheme in the years 2008–2018.

Covariate	Surgical procedure	*N*	Person-years at risk	Cases	IR/95% CI		Crude HR^a^/95% CI		Adjusted^b^ HR(p)/95% CI	
*Total*										
	Both procedures	17,800	90,798	576	6.34	(5.84–6.88)				
	SG	5556	21,035	83	3.95	(3.14–4.89)	1.00			
	RYGB	12,244	69,763	493	7.07	(6.46–7.72)	1.79	(1.42–2.29)	1.69 (<0.001)	(1.33–2.13)
*Sex*										
Men	SG	1405	5626	34	6.04	(4.19–8.45)	1.00			
	RYGB	3270	19,274	187	9.70	(8.36–11.20)	1.61	(1.11–2.39)	1.55 (0.019)	(1.08–2.24)
Women	SG	4151	15,408	49	3.18	(2.35–4.20)	1.00			
	RYGB	8974	50,490	306	6.06	(5.40–6.78)	1.91	(1.41–2.63)	1.78 (<0.001)	(1.31–2.41)
*Age*										
<26 years	SG	412	1622	8	4.93	(2.13–9.72)	1.00			
	RYGB	728	3929	40	10.18	(7.27–13.86)	2.06	(0.95–5.11)	2.03 (0.070)	(0.95–4.35)
26–40 years	SG	1913	7293	29	3.98	(2.66–5.71)	1.00			
	RYGB	4254	25,547	168	6.58	(5.62–7.65)	1.65	(1.11–2.55)	1.51 (0.042)	(1.01–2.25)
>40 years	SG	3231	12,119	46	3.80	(2.78–5.06)	1.00			
	RYGB	7262	40,288	285	7.07	(6.28–7.94)	1.86	(1.36–2.60)	1.74 (<0.001)	(1.27–2.39)

*IR* incidence rate, *HR* hazard ratio, *CI* confidence interval, *RYGB* Roux-n-Y gastric bypass, *SG* sleeve gastrectomy.

^a^SG as reference category.

^b^Adjusted for the covariates sex and/or age.

(*N* = 17,800).

A total of 576 patients met the criteria for AUD+ during the postoperative observation period. Of these, 191 patients had both a diagnosis and a relevant prescription, 349 had a diagnosis only, and 55 received only an AUD-relevant prescription. The overall IR for AUD+ was 6.34 per 1000 person-years for RYGB and SG combined.

### Comparison of operation procedures

**Fig. 1:**
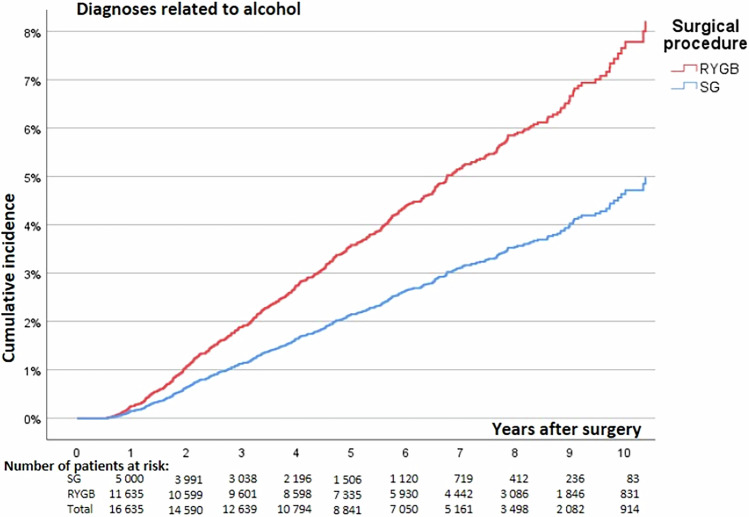
Kaplan–Meier failure curves for time of registration with alcohol-related diagnoses divided by surgical procedure. *RYGB* Roux-en-Y gastric bypass, *SG* sleeve gastrectomy.

The IRs for AUD+ among patients undergoing RYGB were significantly higher than among patients with SG (Table 2). Adjusted HR for age and sex was 1.69 (95% confidence interval (CI) 1.33–2.13, *p* < 0.001) for RYGB compared to SG. The Kaplan–Meier curve in Fig. 1 illustrates the cumulative incidences for AUD+ as time from surgery increases.

In a sensitivity analysis, restricting the cohort to operations performed between January 1, 2014 and December 31, 2018 yielded an age- and sex-adjusted HR for AUD+ after RYGB of 1.70 (95% CI 1.14–2.53) compared to SG.

### Mortality

During the postoperative observation period, in total 262 patients (3.4%) deceased (131 women (1.0%) and 131 men (2.8%)). This corresponds to an overall IR of 2.89 (95% CI 2.55–3.26) per 1000 person years. Of the patients who died, 28 was registered with AUD+. There was no significant difference in mortality between RYGB compared to SG (adjusted HR 1.11, 95% CI 0.82–1.50) in the data available.

For exploring how AUD+ was associated with the risk of death, mortality was calculated independently of surgical procedure. Adjusted HR for dying in the observation period was 2.08 (95% CI 1.40–3.08) for patients with AUD+ compared to those with no such diagnoses.

### Morbidity

The average bariatric surgery patient had 6.7 specialist care contacts (95% CI 6.6–6.9) per year after the surgery. When comparing number of contacts we found significant differences between patients with AUD+ and those with no such diagnoses (F_1,17 798_ = 130.0, *p* < 0.001). On average, patients with no such diagnoses had 6.6 (95% CI 6.4–6.7) contacts per year compared to 11.9 (95% CI 11.2–12.6) contacts for AUD+ patients.

When controlling for possible covariates such as surgical procedure, age and sex, there were significant effects of having alcohol-related diagnoses (+5.5 contacts/year). Of the independent variables, age at the time of surgery had no significant effect on the number of contacts in specialist care. However, both sex (women +1.3) and surgical procedure (RYGB −0.4) showed significant effects. The overall regression model was statistically significant (adjusted R^2^ = 0.017, F_4,17 795_ = 77.1, *p* < 0.001) (Table 3).

**Table 3:** Multiple regressions of number of consultations per year as predicted by alcohol-related diagnoses while controlling for age, sex and surgical procedure (*N* = 17,799).

	No of prescriptions/year		
	*B*	*SE B*	*β*
Constant	6.543	0.314	
Age at time of surgery	−0.016	0.006	−0.020
Sex (female)	1.280	0.148	0.065
Surgical procedure (RYGB)	−0.375	0.140	−0.020
Alcohol-related diagnosis	5.546	0.367	0.113
	Adj. R^2^ = 0.017, *p* < 0.001		

Reference category for surgical procedure is SG.

*RYGB* Roux-en-Y gastric bypass, *SG* sleeve gastrectomy.

We additionally examined morbidity, by the proxies overall prescription counts and defined daily doses after bariatric surgery as exploratory health care utilization measures. These results are presented in Supplementary Material.

## Discussion

In this study we found that patients who had undergone RYGB had 69% higher risk of being diagnosed with a new onset alcohol-related diagnosis compared with those who received SG. Furthermore, these patients had substantially more frequent specialist healthcare contacts and increased mortality risk.

### Alcohol-related diagnoses

This study demonstrates a substantially higher risk of AUD+ following RYGB compared with SG, in line with existing studies comparing these procedures [6, 7]. Mahmud et al., who also collected data on alcohol consumption, found more AUD-related hospital admissions among patients who had undergone RYGB, despite their reporting of lower alcohol intake than SG patients [6]. The authors point to possible explanations related to altered ethanol metabolism after RYGB. Our comparative study of short- and long-term ethanol pharmacokinetics in RYGB and SG provides additional insight: although both procedures nearly double C_max_ and AUC while halving T_max_, these changes are more pronounced with RYGB [17]. While this may not be the only mechanism contributing to increased risk for post-bariatric AUD, the procedure-specific pharmacokinetic differences suggest that RYGB carries a higher risk than SG, in line with our present findings based on this registry data material.

It should be emphasised that our registry endpoint (AUD+) represents clinically recognised alcohol-related morbidity in specialised care and/or reflected by dispensed alcohol-treatment pharmacotherapy, rather than the full spectrum of AUD in the bariatric population. Registry-based ascertainment therefore underestimates true incidence, as many individuals with alcohol-related problems neither reach specialised services (and thus NPR) nor receive pharmacological treatment, and a substantial proportion may not seek healthcare for this at all. Consequently, our incidence estimates should be interpreted as conservative, while the comparative estimates (hazard ratios) remain informative for procedure differences under broadly similar ascertainment mechanisms.

Because RYGB dominated the early years of the dataset, and because we excluded any patient with a documented alcohol diagnosis during the pre-operative look-back period, the main analysis could have exaggerated the HR by preferentially removing high-risk cases from the SG group. To examine this potential calendar-time bias, we ran a sensitivity analysis limited to procedures performed 2014–2018, when both operations were widely used. The age- and sex-adjusted HR for AUD+ after RYGB vs SG in this restricted cohort was essentially identical to the estimate from the full 2008–2018 sample, suggesting that the elevated AUD+ risk is not an artefact of differential baseline exclusions among the earliest RYGB cases.

### Mortality

We observed no difference in mortality between RYGB and SG in the current study. However, our analysis revealed a higher risk of death among AUD+ patients. Even if we did not have access to cause-of-death data in our study, our findings suggest that AUD may be a contributing factor to death in this patient group.

Notably, although mortality due to various cancers, cardiovascular disease, and diabetes declines after RYGB, an increased risk of death from accidents and suicides has been observed compared to individuals with obesity who do not undergo bariatric surgery [22]. Alcohol use may be a contributing factor, given that AUDs is an established risk factor for suicide attempts, and a substantial proportion of such attempts occur under the influence of alcohol [23]. Moreover, AUDs frequently co-occur with other mental disorders, further compounding this risk [24].

Alcohol-related liver disease adds another dimension to the discussion on long-term mortality following bariatric surgery. In a study by Alvarado-Tapias et al. [25], the prevalence of self-reported binge drinking nearly doubled after surgery (from 12% to 23%), and preoperative binge drinking was linked to continued use and elevated risk of death from suicide and liver disease.

Although liver health initially improves after bariatric surgery, postoperative alcohol consumption can negate this benefit. A meta-analysis of severe liver outcomes underscores the importance of extended follow-up, as it may take years to develop alcohol-related cirrhosis and liver cancer [26]. The relatively short follow-up periods in our study (RYGB: 5.7 years; SG: 3.8 years) may be insufficient to capture mortality related to progressive liver disease.

Further, a Belgian study found that patients with alcohol-related cirrhosis who had undergone bariatric surgery were younger, more often female, and exhibited more advanced liver disease despite reporting lower alcohol consumption [27]. These findings suggest that bariatric surgery may create a distinct clinical subgroup–disproportionately vulnerable to alcohol-related harm–whose disease trajectory may be underestimated or missed in routine follow-up and clinical surveillance.

### Morbidity

We observed substantially higher use of specialist healthcare services among patients with AUD+, consistent with an expected overall higher burden of alcohol-related harm. We also explored overall prescription counts as a secondary measure; however, because this outcome is influenced by dispensing practices and package sized, those analyses are reported in the Supplementary Material.

AUD+ patients had, on average, 5.5 more annual healthcare contacts per year compared to patients without alcohol-related diagnoses.

This increase is consistent with well-established findings that alcohol misuse increase the risk of a wide range of illnesses and injuries [28, 29]. In practice, there is a reciprocal influence between the disease burden and the severity of misuse; for instance, a bidirectional relationship between anxiety disorders and alcohol misuse has been documented [30]. Given the serious medical, psychological, and social consequences of substance misuse, both prevention and early intervention might be crucial for improving prognosis.

### Possible implications

Clinicians’ preconceptions about the typical AUD patient may hinder the early detection of alcohol misuse following bariatric surgery. Given that approximately three out of four patients undergoing bariatric surgery are women and the median age is 42 years [31], the demographic profile of those developing de novo AUD postoperatively may differ markedly from the prevailing clinical archetype associated with AUD.

Both RYGB and SG double the systemic exposure to alcohol [17], creating a “stealth effect” whereby harmful alcohol consumption may appear unproblematic–both to patients, their next of kin, and clinicians. Additionally, women may be more vulnerable to alcohol’s adverse effects [32], and a higher age at onset is often associated with more complex comorbidities and an increased risk of drug–alcohol interactions. In older patients, alcohol misuse may also be masked by age-related conditions such as anaemia, liver disease, or falls [33]. Considering that bariatric surgery patients already face an elevated risk of hepatic complications [26, 27], these factors collectively complicate AUD detection.

Particularly in cases of late-onset AUD, we fear that shame and self-stigma may delay recognition and help-seeking, particularly among patients without prior history of alcohol problems who perceive misuse as incongruent with their age or identity. These overlapping vulnerabilities highlight the need for more stringent and proactive alcohol screening protocols than those currently recommended in existing bariatric surgery guidelines.

We observed a higher risk of AUD+ after RYGB compared with SG, consistent with pharmacokinetic evidence [17]. Importantly, this relative difference does not imply that SG is risk-neutral, and patients should be counselled about alcohol-related risks after both procedures. Our findings may nevertheless inform more personalised procedure selection: in patients with multiple risk factors for AUD and no strong contraindication for RYGB, SG may be a preferable option when bariatric surgery is warranted.

### Conclusions and future perspectives

In this nationwide, registry-based cohort study, RYGB was associated with a significantly higher incidence of new onset alcohol-related diagnoses compared to SG. Patients with these diagnoses exhibited markedly increased contacts in specialist care, prescription usage, and a heightened risk of early death which likely undermine the potential health benefits from the operation. Although RYGB was not linked with an overall increase in mortality relative to SG, the burden of alcohol-related diagnoses among patients who have undergone bariatric surgery–especially those receiving RYGB–remains a pressing concern. These findings underscore the importance of improved preoperative screening and postoperative long-term monitoring for alcohol use. Future research should focus on developing effective intervention strategies to mitigate these risks.

### Strengths and limitations

A major strength of this study is the use of nationwide registry data with long-term follow-up, enabling robust incidence calculations and a large, diverse sample. Combining data from two national databases also increased our ability to detect AUD, as patients who had been prescribed medications with high specificity for alcohol problems would be identified, even in the absence of a formal F10 diagnosis. This composite definition also reduces reliance on a single diagnostic code and captures clinically meaningful alcohol-related morbidity through complementary indicators (F10, alcohol-related organ damage, and alcohol-treatment pharmacotherapy). One problem is that we did not include naltrexone, the most frequently used drug for AUD [34]. This was not included because there are also other indications for its use, most notably obesity and chronic fatigue syndrome. However, the observational nature of our design precludes definitive conclusions about causality. Second, registry-based outcomes substantially underestimate true incidence, as most individuals with alcohol-related problems are neither treated in specialised care (not captured in NPR) nor necessarily in primary care (not covered by NPR), and many never seek healthcare at all. To our knowledge, AUD has not been specifically chart-validated in NPR; and even such a validation would mainly assess correct use of F10 among coded cases, not the registry’s ability to capture AUD in the population. Finally, the study would ideally also have included a surgical control group undergoing an operation not expected to alter ethanol kinetics.

## Supplementary information

Supplementary Material

## Data Availability

The data used in this study were obtained from NPR and NorPD under license and ethical approval. Due to legal and privacy restrictions, the individual-level data cannot be shared. Access requires a separate application to the register custodians. Aggregated results are available in the article.
