# Supplementary material for: Alcohol use disorders and related morbidity and mortality after sleeve gastrectomy and Roux-en-Y gastric bypass: a nation-wide registry study (the BAR-REGISTER)
Source: Int J Obes (Lond). 2026 Jun 17;50(7):1581–7. doi: 10.1038/s41366-026-02123-1 (PMC13391372; doi:10.1038/s41366-026-02123-1)
Supplement: Supplementary file 1 — Supplementary Material [file 41366_2026_2123_MOESM1_ESM.docx]

# Supplementary analysis: Dispensed prescriptions and defined daily doses (DDD)

These analyses are presented as Supplementary Material because dispensed prescription data are not necessarily a valid proxy for morbidity, which in the main manuscript is operationalized as number of specialist healthcare consultations. Nonetheless, for exploratory purposes we examined dispensed prescriptions to provide an intuitive description of medication use, while acknowledging that prescription counts may be affected by pack size, strength, and dispensing intervals over time. Therefore, we additionally assessed annual defined daily doses (DDD) as a sensitivity analysis to better capture overall medication volume.

As shown in Supplementary Table, alcohol-related diagnosis was associated with higher medication use across both outcomes after adjustment for age, sex, and surgical procedure (reference category: SG). Alcohol-related diagnosis was associated with 13.6 additional dispensed prescriptions per year and 365 additional DDD per year, corresponding approximately to 1 extra DDD per day. The consistent associations across dispensing frequency (prescriptions/year) and medication volume (DDD/year) suggest that the finding is unlikely to be explained solely by changes in pack size or dispensing patterns and support an interpretation of increased overall medication burden amon patients with alcohol-related diagnoses.

Multiple regressions of annual prescriptions and defined daily doses (DDD) predicted by alcohol-related diagnoses, adjusted for age, sex and surgical procedure (N=17 799). Reference category for surgical procedure is SG.

|  | No of prescriptions/year | | |  | No of DDD/year | | |
| --- | --- | --- | --- | --- | --- | --- | --- |
|  | *B* | *SE B* | *β* |  | *B* | *SE B* | *β* |
| Constant | -2.290 | 0.966 |  |  | -276.196 | 43.591 |  |
| Age at time of surgery | 0.455 | 0.019 | 0.180 |  | 32.785 | 0.841 | 0.282 |
| Sex (female) | 4.803 | 0.455 | 0.078 |  | 104.007 | 20.512 | 0.037 |
| Surgical procedure (RYGB) | 1.805 | 0.430 | 0.031 |  | 85.790 | 19.379 | 0.032 |
| Alcohol diagnosis | 13.645 | 1.128 | 0.089 |  | 365.422 | 50.638 | 0.052 |
|  | Adj. R^2^=0.043, p<0.001 | | |  | Adj. R^2^=0.082, p<0.001 | | |
| Abbreviations: DDD=defined daily doses; RYGB=Roux-en-Y gastric bypass; SG=sleeve gastrectomy | | | | | | | |
